# Chikungunya waves in 2025 signal risk of endemicity in China

**DOI:** 10.1371/journal.pntd.0014685

**Published:** 2026-09-02

**Authors:** Kok Keng Tee, Dongkui Mu, Xueshan Xia

**Affiliations:** Yunnan Provincial Key Laboratory of Public Health and Biosafety, Yunnan Provincial Key Laboratory of Cross-Border Infectious Diseases Control and Drug Innovation, School of Public Health, Kunming Medical University, Yunnan, China; Florida Department of Health, UNITED STATES OF AMERICA

## Abstract

Chikungunya virus (CHIKV) transmission in Guangdong, southern China, intensified markedly in 2025, with successive outbreak waves in Foshan and Jiangmen followed by wider dissemination across all 21 prefectures during a peak domestic travel period. By late 2025, more than 25,000 cases had been reported, suggesting a departure from the historically import-driven and geographically limited outbreaks previously observed in China. These observations raise the possibility of an early transition towards more sustained transmission, although repeated introductions followed by local amplification remain an alternative explanation. Key contributing factors include mobility-associated amplification, climate variability, flood-related expansion of mosquito breeding habitats, favourable urban ecology, and incomplete interruption of transmission during cooler months. Although winter temperatures are suboptimal for sustained transmission, vector persistence and possible vertical viral transmission may permit low-level circulation between epidemic waves. In this context, distinguishing repeated importation from local persistence becomes critical. Preventing endemic establishment will require sustained inter-epidemic surveillance, anticipatory vector control, and clearer policy pathways for deployable vaccination strategies.

## Author summary

In 2025, southern China experienced its largest recorded outbreak of chikungunya, a mosquito-borne viral disease that can cause fever and severe joint pain. Unlike previous outbreaks in China, which were small and linked to imported cases, this outbreak spread widely across an entire province following multiple waves of transmission. We examined this pattern to understand whether it represents a temporary event or an early sign of a more lasting problem.

We propose that several factors may have worked together to drive this unusually large and widespread outbreak. Increased travel during a major national holiday likely helped spread the virus between cities. At the same time, heavy rainfall and flooding may have created more breeding sites for mosquitoes, while urban environments supported ongoing transmission. Even during cooler months, the virus may persist at low levels in mosquito populations.

Taken together, these observations raise the possibility of more sustained local transmission in parts of southern China, although repeated viral introductions remain an alternative explanation. This possibility highlights the need for improved surveillance between outbreaks, better mosquito control strategies, and clearer plans for vaccine use. Recognising these early warning signs is important for preventing long-term establishment of the disease.

**Fig 1:**
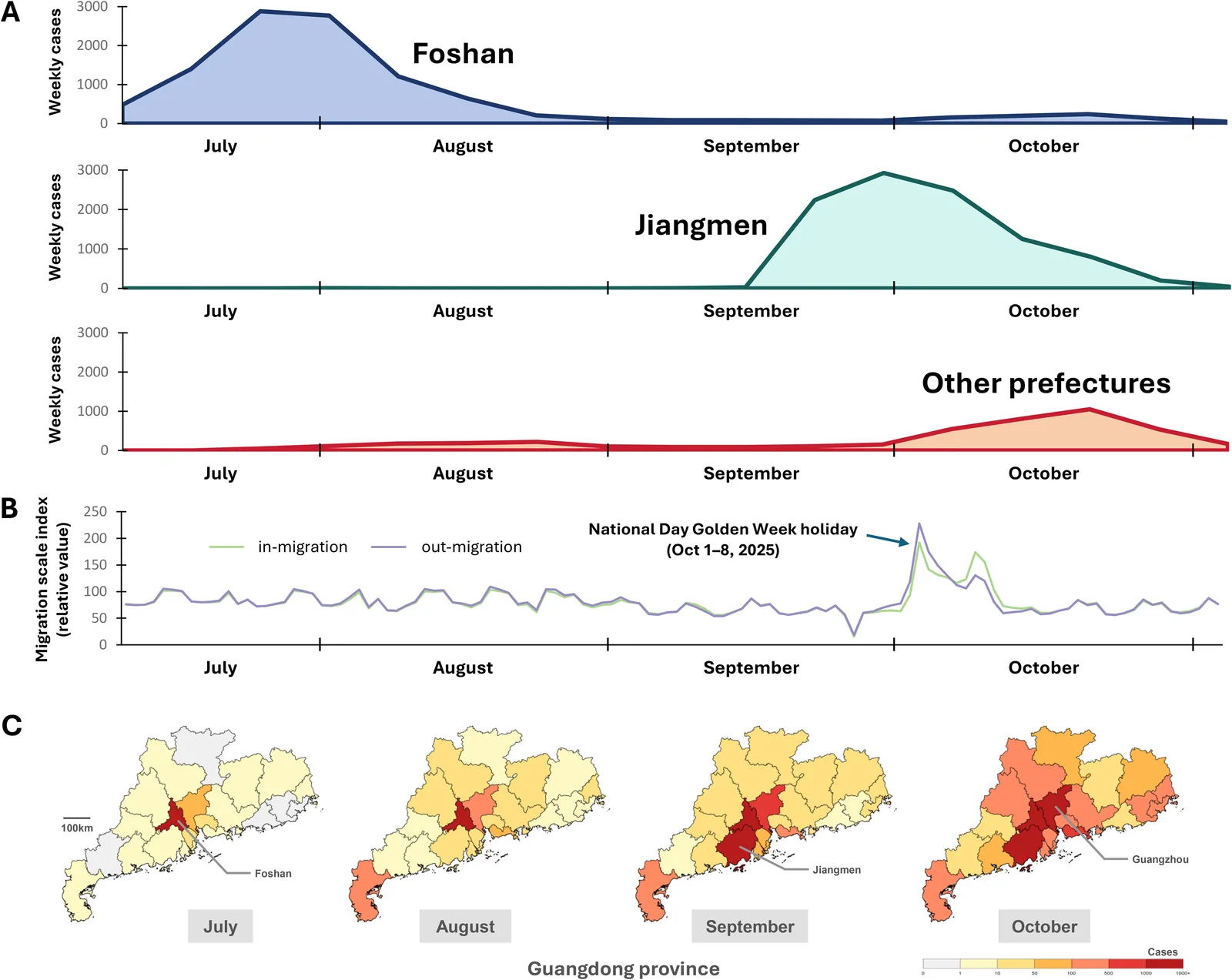
Chikungunya virus outbreak in Guangdong province, China, 2025. **(A)** Weekly PCR-confirmed cases, including symptomatic and asymptomatic infections, in Foshan, Jiangmen, and other prefecture-level cities are shown, revealing three distinct waves of CHIKV transmission peaking in July, September, and October, respectively. The publicly available surveillance reports do not specify whether weekly case attribution is based on symptom onset, specimen collection, laboratory confirmation, or reporting date. Other prefecture-level cities include, in alphabetical order, Chaozhou, Dongguan, Guangzhou, Heyuan, Huizhou, Jieyang, Maoming, Meizhou, Qingyuan, Shantou, Shanwei, Shaoguan, Shenzhen, Yangjiang, Yunfu, Zhanjiang, Zhaoqing, Zhongshan, and Zhuhai. **(B)** Population mobility intensity derived from the Baidu Migration Platform (http://qianxi.baidu.com; accessed 3 November 2025) shows an increase in population movement across the province during early to mid-October, corresponding to the National Day Golden Week holiday (October 1–8, 2025) (S2 Table). The migration scale index represents the relative magnitude of in-migration or out-migration flow, with higher index values indicating greater population mobility. The temporal correspondence between mobility and CHIKV cases is descriptive; no formal statistical association was assessed. **(C)** Geographical distribution and expansion of CHIKV in Guangdong province in July-October 2025. Map Source: (a) Direct link to the map base layer: https://gadm.org/download_country.html. (b) Terms of use/license information: https://gadm.org/license.html.

China reported its largest chikungunya virus (CHIKV) outbreak, which began in Foshan in southern Guangdong province, with more than 8,000 cases recorded between June and August 2025. The public health response, incorporating intensive vector-control measures and community engagement, proved effective and was recognised by WHO as a model for mitigating mosquito-borne diseases [1]. However, Guangdong, the most populous province in China with more than 127 million residents across 21 prefectures, has since experienced an evolving pattern of CHIKV transmission. After initial success in reducing new infections in Foshan and limiting spread to neighbouring prefectures [2], a subsequent outbreak emerged in Jiangmen, south of Foshan, at a comparable scale, peaking in September with nearly 3,000 new weekly cases and resulting in more than 20,000 confirmed cases across the province by late September (Fig 1) [3]. Until then, transmission had remained confined to Foshan and Jiangmen, with limited yet expanding activity observed across other prefectures. However, in early to mid-October, a further increase in CHIKV cases was reported across the remaining 19 prefectures, adding approximately 2,400 new cases (S1 Table). The province-wide surge coincided with the National Day Golden Week holiday (October 1–8, 2025), which overlapped with the Mid-Autumn Festival and represented one of China’s yearly peaks in domestic mobility (Fig 1), likely facilitating broader dissemination of the virus.

Historically, chikungunya fever has imposed a limited burden in China, with outbreaks largely linked to importation. No locally transmitted cases were reported in Guangdong from January to May 2025, with the earliest identified case of the current outbreak having symptom onset on 16 June 2025 [2]. Previous outbreaks were relatively small and geographically localised, including 173 cases in Guangdong in 2010, four cases in Zhejiang in 2017, and 112 cases in Yunnan in 2019 [4–6]. In contrast, the scale and geographic extent of the 2025 Guangdong outbreak—exceeding 25,000 cases across all prefectures—mark a clear departure from previous patterns and raise concern that CHIKV may be approaching sustained circulation in southern China. Understanding this shift requires integrating the roles of human mobility, climatic variability, and vector ecology.

## Mobility-driven amplification of transmission

The October surge coinciding with peak domestic travel highlights human mobility as a key amplifier of arboviral transmission. In Guangdong, increased Golden Week mobility may have facilitated dissemination of CHIKV from initially affected cities to multiple prefectures. However, the temporal relationship between mobility and reported cases should be interpreted cautiously, given delays arising from the intrinsic incubation period in humans, extrinsic incubation in mosquito vectors, and case detection and reporting. Mobile populations can introduce infections into areas with suitable vector densities, enabling rapid local transmission. For *Aedes*-borne viruses, even short-term increases in population mixing can substantially expand outbreak extent, and mobility intensity is strongly associated with the spread and synchronisation of dengue outbreaks across urban settings [7]. Mobility is therefore a predictable and actionable driver of epidemic expansion. Periods of intensified travel represent critical windows for targeted vector control, surveillance, and risk communication. However, sustained transmission still depends on permissive ecological and climatic conditions.

## Climate variability and vector suitability thresholds

Climatic variability also shapes CHIKV amplification and persistence. In southern China, the frequency and intensity of extreme weather events—particularly heavy rainfall and flooding—have increased in recent decades under the combined effects of climate change and urbanisation [8]. In coastal provinces such as Guangdong, monsoonal rainfall and typhoon-associated storms can occur in close succession. Flooding can expand mosquito breeding habitats in urban and peri-urban environments, increasing vector abundance and transmission potential. Recent outbreaks in southern China have coincided with periods of extreme rainfall; in July 2025, rainfall reached 284.7 mm, markedly higher than that recorded in July 2024 (215.9 mm) and July 2023 (156.0 mm), with flood-related water accumulation potentially contributing to increased mosquito breeding and elevated transmission risk [9].

In contrast, seasonal cooling reduces mosquito activity and transmission intensity. Winter temperatures in Guangdong (mean ~15 °C; range 10–22 °C) are suboptimal for sustained transmission by *Aedes aegypti*, although *Aedes albopictus* retains partial activity and greater ecological plasticity in subtropical environments [10,11]. Short-term variability and urban microclimates may still permit intermittent transmission, while climate change is expanding the seasonal window of vector suitability [11]. Together, these dynamics suggest that climatic conditions in southern China may alternately amplify and suppress transmission without fully interrupting circulation.

Seasonal declines in temperature are therefore likely to reduce, but not eliminate, onward transmission. *Ae. albopictus* can overwinter through egg diapause, while *Ae. aegypti* survives mainly through short-term quiescence of desiccation-resistant eggs. Vertical transmission of CHIKV in Aedes mosquitoes provides an additional mechanism for persistence during unfavourable periods and re-emergence when conditions become permissive [10]. Transmission may therefore continue at low levels between epidemic waves.

## Importation versus local persistence: An attribution challenge

A key question is whether the observed transmission reflects repeated viral importations or the emergence of sustained local persistence. Importantly, the epidemiological patterns observed in Guangdong alone cannot distinguish between these possibilities. In highly connected settings, repeated introductions into receptive environments can generate recurrent outbreaks that resemble sustained local transmission. Thus, the multiple transmission waves observed in 2025 should be regarded as a potential warning signal rather than evidence of endemic establishment. Case-based surveillance alone is often insufficient to distinguish between these scenarios, particularly during inter-epidemic periods. Genomic epidemiology is critical for distinguishing repeated introductions from sustained local circulation [10]. Evidence of endemic establishment would include phylogenetic continuity and persistence of locally circulating lineages across seasons, with fewer independent introductions. Recent genomic studies in China indicate multiple introductions followed by substantial local transmission, but no evidence of long-term sustained circulation [12,13]. Systematic genomic surveillance across epidemic and inter-epidemic periods will therefore be essential to detect persistent local lineages.

This attribution is particularly challenging given heightened global CHIKV activity in 2025, with more than 445,000 suspected and confirmed cases reported across 40 countries by September [14]. Increased international transmission may have increased importation pressure into China, while subsequent domestic mobility could have facilitated local amplification and dissemination. Thus, the observed transmission waves may reflect repeated introductions and domestic spread rather than continuous local persistence alone.

## From epidemic waves to endemic transition

The Guangdong pattern—sequential localised outbreaks followed by province-wide synchronisation (Fig 1)—raises the hypothesis that conditions may be emerging that could support an endemic transition. CHIKV establishment can be conceptualised as a multistage process: (i) importation-driven outbreaks, (ii) amplification under favourable conditions, (iii) inter-epidemic persistence, and (iv) stabilisation into seasonal or endemic transmission. Multiple transmission waves within a single year, together with expansion across all prefectures, are compatible with, but do not demonstrate, progression towards such a transition. Alternative explanations, particularly repeated viral introductions followed by local amplification, remain plausible. Comparable trajectories have been documented in the Indian Ocean region and the Americas, where large epidemics were followed by recurrent outbreaks and, in some settings, sustained transmission over time [15,16]. Because this transition is often recognised only retrospectively, early warning signals are critical. Taken together, these patterns suggest that the 2025 Guangdong outbreak may represent an early signal of endemic transition.

## Inter-epidemic surveillance as a critical blind spot

If such a transition is underway, detecting transmission between epidemic waves becomes critical. Current surveillance systems are optimised for outbreak detection rather than for characterising low-level inter-epidemic transmission. As a result, persistence, whether maintained through low-level human transmission or vector-mediated mechanisms such as vertical transmission, remains poorly understood. For arboviruses, apparent absence of reported cases may reflect under-detection rather than true interruption. Strengthening inter-epidemic surveillance through integrated clinical, entomological, and genomic approaches will therefore be essential both to detect early signals of endemic establishment and to distinguish persistent local transmission from repeated importation.

## Urban ecology and vector adaptation

In rapidly urbanising areas of Guangdong and southern China, environmental modification has created habitats highly favourable for *Ae. albopictus*, including peri-urban expansion, construction-associated water accumulation, and dense human-vector contact. Unlike *Ae. aegypti*, which is more restricted to tropical climates, *Ae. albopictus* tolerates broader environmental conditions and has been implicated in CHIKV transmission in temperate regions [17]. Its ecological flexibility increases the likelihood that introduced infections can be maintained locally, increasing the potential for introduced infections to generate prolonged or recurrent local transmission.

## The arboviral vaccine gap and implications for CHIKV control

As the risk of sustained CHIKV circulation increases, limited availability and deployment of chikungunya vaccines pose an additional constraint on long-term control. Early deployment has been largely confined to high-income settings and travel-related use, with little integration into routine immunisation programmes in at-risk regions. This reflects a broader disconnect between vaccine availability and effective protection, whereby regulatory approval does not necessarily translate into timely or equitable access [10,18].

In southern China, this reinforces reliance on vector control and surveillance as primary interventions. Without clear deployment frameworks and policy pathways, newly available vaccines are unlikely to meaningfully alter transmission dynamics. This becomes more consequential if CHIKV progresses towards sustained circulation. While reactive vaccination may have limited impact during large outbreaks, proactive or targeted deployment could reduce transmission intensity or prevent establishment in high-risk settings. Without such strategies, vector control alone may be insufficient to prevent endemic stabilisation, particularly in settings characterised by high population density, environmental suitability, and recurrent amplification events.

## Conclusion

The Guangdong outbreak in 2025 raises the possibility of an early transition towards more sustained CHIKV transmission, although repeated introductions followed by local amplification remain an alternative explanation. The convergence of mobility-driven dissemination, climate variability, vector adaptation, and incomplete interruption of transmission creates conditions favourable for sustained circulation, but evidence of inter-epidemic persistence is currently lacking. Reliance on episodic control measures is therefore unlikely to be sufficient. Preventing endemic establishment will require sustained inter-epidemic surveillance, anticipatory vector control, and deployable vaccination strategies aligned with evolving transmission risk.

### Ethical approval statement

Not applicable, as this study was based on publicly available data and did not involve human participants, animals, or the use of identifiable personal data.

## Supporting information

S1 Table Distribution of chikungunya virus (CHIKV) infections across 21 prefectures in Guangdong Province, China, 2025. (DOCX)

S2 Table Migration scale index during the National Day Golden Week holiday in Guangdong, October 1–8, 2025. (DOCX)
