# Supplementary material for: Chikungunya waves in 2025 signal risk of endemicity in China
Source: PLoS Negl Trop Dis. 2026 Sep 2;20(9):e0014685. doi: 10.1371/journal.pntd.0014685 (PMC13537560; doi:10.1371/journal.pntd.0014685)
Supplement: Supplementary file 1 [file pntd.0014685.s001.docx]

**S1 Table.** Distribution of chikungunya virus (CHIKV) infections across 21 prefectures in Guangdong Province, China, 2025.

| Date | Foshan | Jiangmen | Chaozhou | Dongguan | Guangzhou | Heyuan | Huizhou | Jieyang | Maoming | Meizhou | Qingyuan | Shantou | Shanwei | Shaoguan | Shenzhen | Yangjiang | Yunfu | Zhanjiang | Zhaoqing | Zhongshan | Zhuhai |
| --- | --- | --- | --- | --- | --- | --- | --- | --- | --- | --- | --- | --- | --- | --- | --- | --- | --- | --- | --- | --- | --- |
| Jul 8-15 | 481 | 0 | 0 | 0 | 0 | 0 | 0 | 0 | 0 | 0 | 0 | 0 | 0 | 0 | 0 | 0 | 0 | 0 | 0 | 0 | 0 |
| Jul 16-19 | 1403 | 0 | 0 | 0 | 0 | 0 | 0 | 0 | 0 | 0 | 0 | 0 | 0 | 0 | 0 | 0 | 0 | 0 | 0 | 0 | 0 |
| Jul 20-26 | 2882 | 2 | 0 | 3 | 22 | 3 | 0 | 0 | 0 | 0 | 1 | 0 | 0 | 0 | 1 | 2 | 0 | 1 | 2 | 18 | 3 |
| Jul 27-Aug 2 | 2770 | 6 | 2 | 11 | 65 | 0 | 4 | 0 | 0 | 5 | 0 | 0 | 0 | 0 | 6 | 1 | 1 | 0 | 6 | 11 | 4 |
| Aug 3-9 | 1212 | 1 | 1 | 2 | 103 | 0 | 3 | 1 | 0 | 0 | 6 | 1 | 0 | 2 | 5 | 1 | 1 | 39 | 0 | 4 | 5 |
| Aug 10-16 | 644 | 2 | 9 | 7 | 85 | 2 | 16 | 0 | 0 | 2 | 5 | 0 | 0 | 0 | 16 | 1 | 4 | 22 | 1 | 4 | 10 |
| Aug 17-23 | 206 | 1 | 13 | 5 | 62 | 8 | 6 | 1 | 1 | 1 | 0 | 0 | 0 | 2 | 37 | 3 | 0 | 77 | 0 | 0 | 3 |
| Aug 24-30 | 119 | 2 | 5 | 2 | 49 | 0 | 1 | 0 | 0 | 1 | 2 | 3 | 1 | 1 | 16 | 0 | 1 | 14 | 1 | 4 | 3 |
| Aug 31-Sep 6 | 85 | 4 | 2 | 3 | 40 | 2 | 1 | 0 | 0 | 0 | 3 | 2 | 0 | 1 | 21 | 0 | 3 | 4 | 0 | 2 | 5 |
| Sep 7-13 | 86 | 26 | 1 | 2 | 41 | 1 | 5 | 0 | 1 | 2 | 1 | 5 | 1 | 1 | 19 | 0 | 0 | 4 | 1 | 1 | 4 |
| Sep 14-20 | 81 | 2238 | 3 | 5 | 47 | 0 | 1 | 1 | 1 | 1 | 4 | 3 | 1 | 5 | 12 | 1 | 0 | 7 | 3 | 6 | 7 |
| Sep 21-27 | 78 | 2927 | 3 | 7 | 68 | 0 | 1 | 0 | 1 | 3 | 6 | 4 | 1 | 3 | 22 | 1 | 1 | 6 | 5 | 3 | 13 |
| Sep 28-Oct 4 | 157 | 2480 | 17 | 28 | 138 | 3 | 4 | 7 | 8 | 7 | 12 | 51 | 3 | 7 | 81 | 10 | 6 | 22 | 101 | 18 | 21 |
| Oct 5-11 | 196 | 1255 | 38 | 37 | 201 | 4 | 26 | 44 | 19 | 19 | 34 | 38 | 16 | 19 | 109 | 18 | 15 | 23 | 58 | 35 | 53 |
| Oct 12-18 | 241 | 795 | 81 | 41 | 189 | 10 | 29 | 89 | 8 | 29 | 39 | 68 | 13 | 27 | 202 | 40 | 10 | 13 | 57 | 52 | 53 |
| Oct 19-25 | 130 | 199 | 32 | 33 | 124 | 6 | 11 | 20 | 0 | 5 | 16 | 43 | 2 | 19 | 102 | 12 | 2 | 16 | 33 | 30 | 33 |
| Oct 26-Nov 1 | 51 | 44 | 10 | 9 | 37 | 1 | 5 | 3 | 1 | 3 | 0 | 3 | 0 | 0 | 48 | 0 | 0 | 5 | 8 | 9 | 18 |
| Nov 2-8 | 21 | 23 | 2 | 3 | 8 | 0 | 0 | 1 | 0 | 0 | 0 | 0 | 0 | 0 | 30 | 0 | 0 | 3 | 1 | 5 | 10 |
| Nov 9-15 | 8 | 14 | 0 | 1 | 4 | 0 | 1 | 0 | 0 | 0 | 0 | 0 | 0 | 0 | 12 | 0 | 0 | 0 | 0 | 4 | 4 |
| Nov 16-22 | 9 | 8 | 1 | 2 | 4 | 0 | 0 | 0 | 0 | 0 | 0 | 0 | 0 | 0 | 11 | 0 | 0 | 0 | 1 | 1 | 6 |
| Nov 23-29 | 2 | 6 | 0 | 0 | 2 | 0 | 0 | 0 | 0 | 0 | 0 | 3 | 0 | 0 | 12 | 0 | 0 | 1 | 0 | 0 | 1 |
| Nov 30-Dec 6 | 1 | 1 | 0 | 1 | 1 | 0 | 0 | 0 | 0 | 0 | 0 | 0 | 0 | 0 | 4 | 0 | 0 | 0 | 0 | 1 | 0 |
| Dec 7-13 | 1 | 0 | 0 | 0 | 2 | 0 | 0 | 0 | 0 | 0 | 0 | 1 | 0 | 0 | 5 | 0 | 0 | 0 | 0 | 0 | 0 |
| Dec 14-20 | 0 | 1 | 0 | 0 | 0 | 0 | 1 | 0 | 0 | 0 | 0 | 0 | 0 | 0 | 0 | 0 | 0 | 0 | 0 | 0 | 0 |
| Dec 21-27 | 0 | 0 | 0 | 0 | 0 | 0 | 0 | 0 | 0 | 0 | 0 | 0 | 0 | 0 | 0 | 0 | 0 | 0 | 0 | 0 | 0 |

Source: Guangdong Provincial Center for Disease Control and Prevention. Epidemic Information 2025 (available from: https://cdcp.gd.gov.cn/ywdt/zdzt/yfjkkyr/yqxx/) (accessed 31 December 2025
