# Supplementary material for: Chikungunya waves in 2025 signal risk of endemicity in China
Source: PLoS Negl Trop Dis. 2026 Sep 2;20(9):e0014685. doi: 10.1371/journal.pntd.0014685 (PMC13537560; doi:10.1371/journal.pntd.0014685)
Supplement: Supplementary file 2 [file pntd.0014685.s002.docx]

**S2 Table.** Migration scale index during the National Day Golden Week holiday in Guangdong, October 1–8, 2025.

| Prefecture-level city | Movement type | Baseline (based on September average) | Golden Week | Change | Change (%) |
| --- | --- | --- | --- | --- | --- |
| Chaozhou | In-migration | 0.83922048 | 2.223357429 | 1.384136949 | 164.93 |
|  | Out-migration | 0.77271408 | 1.821764057 | 1.049049977 | 135.76 |
| Dongguan | In-migration | 7.74485388 | 13.91991943 | 6.175065549 | 79.73 |
|  | Out-migration | 8.70937704 | 18.2581776 | 9.54880056 | 109.64 |
| Foshan | In-migration | 7.59006828 | 13.28823514 | 5.698166863 | 75.07 |
|  | Out-migration | 8.18713872 | 15.53496223 | 7.347823509 | 89.75 |
| Guangzhou | In-migration | 12.24170712 | 21.10689669 | 8.865189566 | 72.42 |
|  | Out-migration | 12.07658268 | 25.77527383 | 13.69869115 | 113.43 |
| Heyuan | In-migration | 0.84878604 | 3.972651943 | 3.123865903 | 368.04 |
|  | Out-migration | 0.76010832 | 3.072741943 | 2.312633623 | 304.25 |
| Huizhou | In-migration | 4.75055928 | 11.06709017 | 6.316530891 | 132.96 |
|  | Out-migration | 4.75493868 | 10.12222749 | 5.367288806 | 112.88 |
| Jiangmen | In-migration | 2.04990372 | 4.691626457 | 2.641722737 | 128.87 |
|  | Out-migration | 1.99322532 | 4.117021714 | 2.123796394 | 106.55 |
| Jieyang | In-migration | 1.31630292 | 3.648698229 | 2.332395309 | 177.19 |
|  | Out-migration | 1.29700332 | 3.085725086 | 1.788721766 | 137.91 |
| Maoming | In-migration | 1.15636896 | 4.336698343 | 3.180329383 | 275.03 |
|  | Out-migration | 1.14963624 | 3.631595657 | 2.481959417 | 215.89 |
| Meizhou | In-migration | 0.747198 | 3.550952057 | 2.803754057 | 375.24 |
|  | Out-migration | 0.65889612 | 3.082336971 | 2.423440851 | 367.8 |
| Qingyuan | In-migration | 1.94136696 | 7.770390171 | 5.829023211 | 300.25 |
|  | Out-migration | 1.5862554 | 4.315568914 | 2.729313514 | 172.06 |
| Shantou | In-migration | 1.47825864 | 3.796743086 | 2.318484446 | 156.84 |
|  | Out-migration | 1.53326304 | 3.744657771 | 2.211394731 | 144.23 |
| Shanwei | In-migration | 0.79293492 | 2.592124971 | 1.799190051 | 226.9 |
|  | Out-migration | 0.74733084 | 1.813904743 | 1.066573903 | 142.72 |
| Shaoguan | In-migration | 0.78120288 | 3.209594914 | 2.428392034 | 310.85 |
|  | Out-migration | 0.70297416 | 2.6445528 | 1.94157864 | 276.19 |
| Shenzhen | In-migration | 9.1658034 | 17.48558109 | 8.319777686 | 90.77 |
|  | Out-migration | 10.19076552 | 22.66844709 | 12.47768157 | 122.44 |
| Yangjiang | In-migration | 0.66143628 | 2.438664686 | 1.777228406 | 268.69 |
|  | Out-migration | 0.626616 | 1.860588514 | 1.233972514 | 196.93 |
| Yunfu | In-migration | 0.71589204 | 3.012876 | 2.29698396 | 320.86 |
|  | Out-migration | 0.68328252 | 1.918038343 | 1.234755823 | 180.71 |
| Zhanjiang | In-migration | 1.29605616 | 3.669258343 | 2.373202183 | 183.11 |
|  | Out-migration | 1.19866608 | 3.242629029 | 2.043962949 | 170.52 |
| Zhaoqing | In-migration | 1.79769132 | 6.545304514 | 4.747613194 | 264.1 |
|  | Out-migration | 1.61221212 | 4.129426286 | 2.517214166 | 156.13 |
| Zhongshan | In-migration | 3.84998724 | 6.522212571 | 2.672225331 | 69.41 |
|  | Out-migration | 4.19112036 | 7.7762592 | 3.58513884 | 85.54 |
| Zhuhai | In-migration | 2.82704904 | 4.673237143 | 1.846188103 | 65.3 |
|  | Out-migration | 2.69838 | 4.592778686 | 1.894398686 | 70.21 |

Source: Baidu Migration Platform (available from: http://qianxi.baidu.com) (accessed 3 November 2025)
